# Effectiveness of Adapted Physical Activity on Quality of Life of Patients with Knee and Hip Replacement: A Randomized Pilot Study

**DOI:** 10.3390/healthcare13182333

**Published:** 2025-09-17

**Authors:** Raffaele Zinno, Erika Pinelli, Giuseppe Barone, Dante Dallari, Maria Scoppolini Massini, Laura Bragonzoni

**Affiliations:** 1 Department for Life Quality Studies, University of Bologna, 47921 Rimini, Italy; erika.pinelli2@unibo.it (E.P.); giuseppe.barone8@unibo.it (G.B.); maria.scoppolini2@unibo.it (M.S.M.); laura.bragonzoni4@unibo.it (L.B.); 2 Reconstructive Orthopaedic Surgery and Innovative Techniques—Musculoskeletal Tissue Bank, IRCCS Rizzoli Orthopedic Institute, 40136 Bologna, Italy; dante.dallari@ior.it

**Keywords:** total joint replacement, functional test, pain, lower limb strength, exercise adherence

## Abstract

**Background:** Total hip (THR) and knee replacement (TKR) effectively treat end-stage osteoarthritis, but many patients continue to experience functional limitations and reduced quality of life (QoL) after rehabilitation. The aim of this pilot study was to assess the changes in terms of QoL in people with THR and TKR after a specifically designed PA intervention. The secondary aim was to evaluate changes in physical function through strength and mobility tests. **Methods**: Eighteen participants (mean age 65.8 ± 7.1 years) were enrolled at the Rizzoli Orthopedic Institute and were randomly assigned to an intervention group (IG), which completed a six-month supervised PA program (6 months after surgery), or a control group (CG), which received standard care. Assessments were conducted at four time points: before surgery, after rehabilitation, and at 9- and 12-month post-surgery. Repeated measures ANOVA was used to assess within- and between-group differences over time, with post hoc pairwise comparisons conducted using independent *t*-tests with Sidak correction. The level of statistical significance was set at *p* < 0.05 for all analyses. **Results:** Both groups showed significant improvements in QoL over time, with greater gains in physical functioning observed in the IG. Lower limb strength increased more in the IG; however, the differences between groups were not statistically significant. The Timed Up and Go and 30-Second Chair Stand Test improved in both groups. No adverse events were reported. **Conclusions:** These findings support the feasibility and potential benefits of adapted PA programs after rehabilitation. Although no significant differences emerged between groups, clinically relevant improvements were observed in the IG. Larger studies are warranted to confirm these results and explore long-term outcomes across multiple domains.

## 1. Introduction

Osteoarthritis (OA) is a common joint disorder, and its incidence is rising as the world’s population ages [1]. Clinical symptoms of OA include pain, brief morning stiffness, and crepitus during joint mobility, all of which significantly reduce daily quality of life (QoL) [2,3]. Moreover, OA can result in a variety of personal, social, and economic consequences, including decreased productivity, early retirement from the workforce, loss of independence, the need for assistance, and increased use of health services [4,5,6].

Nowadays, the preferred treatment for end stage OA is total joint replacement since the surgical treatment can alleviate the pain [7,8,9] and thereby improve QoL. The multidimensional concept of QoL includes personal health (physical, mental, and spiritual), relationships, education status and social status, work environment, a sense of security and safety, and autonomy. These elements, especially physical and mental [10], are negatively influenced by OA, which most commonly affects the hip and knee [4], often leading to total hip and knee replacement (THR and TKR), the most frequently performed surgical procedures [11,12,13,14]. However, such surgical procedures alone may not fully restore the natural joint function [15,16] and patients report dissatisfaction rates of about 10% for THR and 15–30% for TKR [17].

According to the Organization for Economic Cooperation and Development (OECD), there were 193.9 THR and 137.0 TKR cases per 100,000 people in Italy [18]. These numbers are relatively consistent with OECD country averages for 2019 (174.1 THR and 136.7 TKR per 100,000 people [18]). There is consensus that people with THR and TKR can experience several benefits from being physically active, including a reduced risk of falls, increased bone density, improved prosthetic fixation, and decreased risk of prosthetic loosening [19,20]. According to the World Health Organization (WHO), adults aged 65 and older should aim for at least 150 min per week of moderate-intensity aerobic activity (or 75 min per week of vigorous activity), with strength training on two or more days per week [21]. Moreover, increased physical activity (PA) is strongly associated with improved quality of life (QoL) across age groups and populations [22]. Despite the advantages of postoperative PA program following TKR and THR [23] and the proposal of various PA interventions for people with total joint replacement, the existing heterogeneity in study designs and outcome measures, make difficult to draw definitive conclusions or establish universal protocols [24] concerning the type, setting, frequency, intensity, and duration of therapy. Moreover, the relationship between activity levels and implant failure in TKR remains unclear [25]. Randomized controlled trials (RCTs) are fundamental to assess the effectiveness of PA interventions and to provide evidence and clinical insights for individuals with THR and TKR. While several RCTs have been conducted among this population to improve strength, physical function, clinical outcomes [26,27,28,29,30], many of them have primarily focused on pre surgery [27,30] or early rehabilitation phases [26,28,29,30] with limited emphasis on the post rehabilitation period, which is critical for long-term recovery.

Although early rehabilitation phase following surgery plays a vital role in restoring joint function, overall outcome, and joint mobility [29,31], the post-rehabilitation period plays a key role in maintaining gains, preventing relapse into inactivity, and promoting an active lifestyle. However, the type and duration of the rehabilitation may vary significantly, influenced by national guidelines (if available) and clinic and therapist protocols. Unfortunately, a post rehabilitation exercise program often receives insufficient attention, despite their potential to promote an active lifestyle and enhance physical benefits.

Consequently, individuals may return to inactivity after completing the early rehabilitation. Therefore, supervised and adapted exercise programs beyond the early rehabilitation phase are essential to consolidate functional gains, enhance fitness, and support long-term joint health, while considering individual limitations to minimize the risk of injury.

The aim of this pilot study was to assess the changes in terms of QoL in people with THR and TKR after a specifically designed PA intervention. The secondary aim was to evaluate changes in physical function through strength and mobility tests. We hypothesize that participants with THR or TKR who engage in a specifically designed post-rehabilitation PA intervention will demonstrate a statistically significant and clinically meaningful improvement in QoL compared to those receiving standard care.

## 2. Materials and Methods

The “Physical ActIvity after hip and knee Replacement” (PAIR) project, funded within the Erasmus Plus Sport program (Grant Agreement 613008-EPP-1-2019-1-IT-SPO-SCP), conceived this pilot study. The study received approval from the Local Ethics Committee (Comitato Etico Indipendente di Area Vasta Emilia Centro, CE-AVEC) of the Emilia-Romagna Region (reference number AVEC: 1005/2020/Sper/IOR) and is registered on ClinicalTrial.Gov (NCT04761367). A detailed description of the methods is provided in a previous protocol study [32]. In brief, this single-blind randomized controlled pilot trial, with researchers performing the assessments blinded to group allocation, was designed to evaluate the effectiveness of a tailored exercise program in individuals following THR and TKR.

### 2.1. Recruitment and Randomization Process

The total number of patients with THR and TKR identified to conduct the large-scale RCT was 80, according to a priori power analysis calculated on the study’s primary outcome [32]. This pilot study reports preliminary results from a cohort of 18 patients with a mean age of 65.8 ± 7.1. Patients were recruited during their pre-surgery medical check-up, where they signed the informed consent form. Each patient underwent either TKR (*n* = 4) or THR (*n* = 14). Six months after surgical treatment and subsequent to the standard rehabilitation, participants were randomly assigned to one of two groups: Intervention Group (IG) and Control Group (CG). Randomization was performed using block randomization to ensure balanced group sizes throughout the study. The CG consisted of 11 participants (10 with THR and 1 with TKR), while the IG included 7 participants (4 with THR and 3 with TKR). The IG followed a 6-month exercise program specifically designed for people with knee and hip arthroplasty and received educational sessions on the importance of an active lifestyle. In contrast, the CG received the standard care recommended for such patients by surgeons and healthcare professionals. Standard care was coordinated among the orthopedic surgeon, physiatrist, and physical therapist, and adapted individually based on patient condition. The program included two daily sessions of gradually increasing difficulty and simple exercises that patients were instructed to perform independently several times per day. Discharge typically occurred 4–5 days after surgery. Patients were advised to continue the exercises learned during hospitalization at home and, depending on joint condition, to continue specific rehabilitation at a dedicated center.

**Figure 1:**
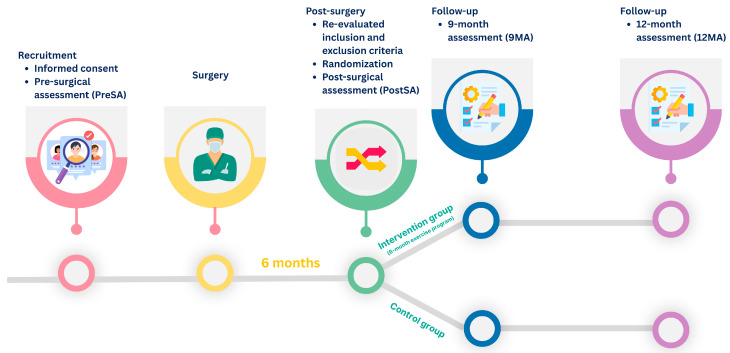
Study timeline and assessment schedule.

Both groups were assessed at four time points (Figure 1):

(1) within 2-week before surgery, corresponding to the pre-surgical assessment (PreSA);
(2) six months after surgery (PostSA);
(3) three months after the exercise program, corresponding to nine months after surgery (9MA);
(4) six months after the exercise program, corresponding to 12 months after surgery (12MA).

Patients were asked to participate in the study during their PreSA and the patients signed the informed consent form. The recruitment procedure was carried out by the medical personnel of the Clinical Units Chirurgia Ortopedica Ricostruttiva Tecniche Innovative and Clinica Ortopedica e Traumatologica II of the Istituto Ortopedico Rizzoli of Bologna.

### 2.2. Inclusion and Exclusion Criteria

The inclusion and exclusion criteria were assessed two different times: during the PreSA and PostSA, which correspond to the end of the rehabilitation phase. The inclusion criteria were: signed informed consent, age between 50 and 80 years, advanced unilateral osteoarthritis requiring primary THR or TKR, and the American Society of Anesthesiologists classification between 1 and 2 levels. The exclusion criteria were: (1) Unable/unwilling to sign the informed consent form of the study and/or willing to comply with the study requests; (2) Poor knowledge of Italian language which prevents understanding of the content of the consent form and/or of instructions for assessment and/or training; (3) Severe functional limitations of other lower extremity joints besides that for which surgery is planned; (4) Impairment of communicative and/or sensory functions so severe to make impossible understanding or executing trainer’s instructions (dementia, aphasia, blindness, deafness); (5) Heart failure (NYHA 2 class > 2); (6) Unstable angina; (7) Pulmonary disease requiring oxygen therapy; (8) Symptomatic peripheral arteriopathy; (9) Recent myocardial infarction or hospital admission for any other reason in the previous six months; (10) Symptomatic orthostatic hypotension; (11). Hypertension in poor pharmacologic control (diastolic > 95 mmHg, systolic > 160 mmHg); (12) Relevant neurological condition impairing motor or cognitive function; (13) Any other condition that the medical doctor considers contraindicating the participation in an exercise program of moderate intensity; (14) Severe depression.

During PostSA, two additional exclusion criteria were checked: (1) Functional performance: Able to stand and walk > 500 m independently; (2) Pain: Visual Analogue Scale (VAS) score ≥ 4 during rest.

### 2.3. Exercise Program for Intervention Group

Two distinct, tailored exercise programs were developed for individuals with THR and TKR. These programs were designed in accordance with the WHO guidelines on PA for older and the clinical recommendations of orthopedic surgeons regarding PA after joint replacement [19]. All activities were gym-based and supervised by a qualified exercise specialist experienced in working with post-surgical populations. The content and progression of the programs were based on the findings of systematic reviews and grounded in clinical reasoning by experts in exercise specialist, rehabilitation medicine, and physical therapy. The primary goals of both programs were to enhance postural control, proprioception, muscular strength, joint range of motion, and cardiovascular endurance. To ensure safety, the programs excluded movements that could compromise prosthesis integrity, such as kneeling for individuals after TKR and excessive hip adduction or flexion for individuals after THR, consistent with current post-operative precautions [8,19].

Each exercise session followed a specific structure comprising three phases: initiation (warm-up), conditioning (balance and strength exercises), and cooling down and stretching. Table 1 provide a single representative example of an exercise session.

### 2.4. Outcomes

The primary outcome of this study was health-related QoL, assessed using the 36-Item Short Form Survey (SF-36) [33], which evaluates eight health domains: physical functioning, role limitations due to physical health, role limitations due to emotional problems, energy/fatigue, emotional well-being, social functioning, pain, and general health, along with a health change score [34]. The secondary outcomes included a combination of patient-reported outcome measures (PROMs), objective physical assessments, and functional tests.

PROMs comprised: The VAS for pain intensity [35,36], the Western Ontario and McMaster Universities Osteoarthritis Index (WOMAC) for evaluating pain, stiffness, and physical function in patients with hip and knee OA [37], the Hip disability and Osteoarthritis Outcome Score (HOOS) [38] and the Knee injury and Osteoarthritis Outcome Score (KOOS) [39], which are joint-specific tools assessing symptoms, pain, function, and QoL related to hip and knee arthroplasty, respectively.

Additionally, objective strength measures were obtained using a handheld dynamometer [40], focusing on hip flexion, extension, abduction, and adduction of both the operated and non-operated limbs, as well as knee flexion [41].

Functional performance was assessed using three validated tests: Timed Up and Go (TUG) test, which measures basic functional mobility and fall risk [42], 30-Second Chair Stand Test (30sCST), which evaluates lower-body strength [43], and Single Stance Test, which assesses static balance and postural control.

Finally, the adherence to the exercise program of the IG was recorded during each session by the trainer.

Table 2 provides a summary of the instruments used, including their administration method and their main purpose.

### 2.5. Statistics

All statistical analyses were conducted using Python (version 3.11.11) within the Spyder development environment. Descriptive statistics were reported as mean and standard deviation (SD) for normally distributed variables, and as median and interquartile range (IQR) for non-normally distributed variables.

When normality was confirmed, parametric tests were applied. The assumption of homogeneity of variances was assessed using Bartlett’s test, with a significance threshold of *p* < 0.01. If variances were unequal, Welch’s *t*-test was used; otherwise, the standard Student’s *t*-test was performed. The Mann–Whitney was used for non-parametric variables. The Chi-squared tests were used to assess differences in categorical variables (Surgery type and side, gender, educational level, occupation, and family status). Repeated measures ANOVA were performed to evaluate within-group and between-group differences across time points for each outcome variable. The assumption of sphericity was tested using Mauchly’s test; when violated, the Greenhouse–Geisser correction was applied. Pairwise post hoc comparisons were conducted using independent *t*-tests with Sidak correction for multiple comparisons. The level of statistical significance was set at *p* < 0.05 for all analyses.

## 3. Results

The comparison of the sample characteristics did not show differences between CG and IG (Table 3).

### 3.1. Quality of Life

**Figure 2:**
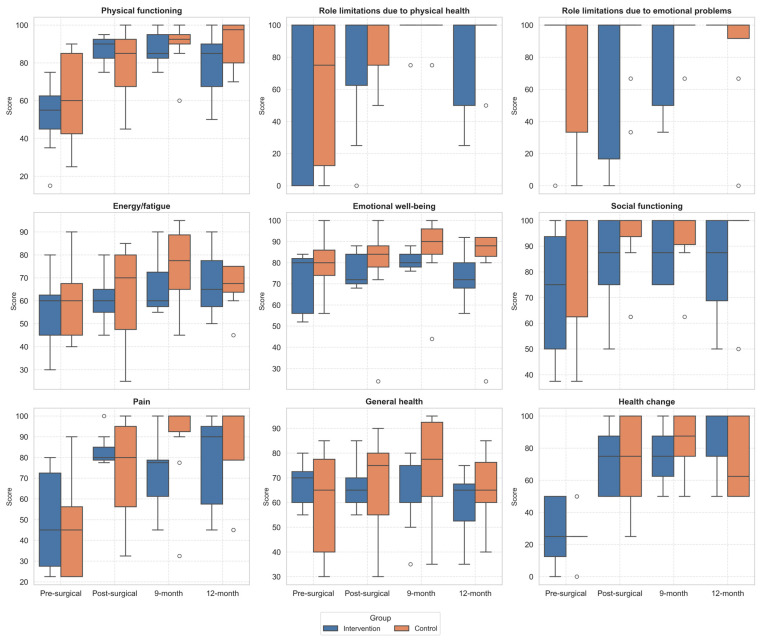
Boxplot of changes in SF36 domains across follow-up assessments. Outliers, values outside the range of 1.5*IQR, are shown as circles.

The analysis of the SF-36 results showed that both groups experienced improvements in different domains over the follow-up period (Table 4, Figure 2). The repeated measures ANOVA showed significant within-subject effects over time for physical functioning (*p* < 0.001, η^2^ = 0.53), pain (*p* = 0.001, η^2^ = 0.38), health change (*p* < 0.001, η^2^ = 0.53), and energy/fatigue (*p* = 0.028, η^2^ = 0.22), suggesting an overall improvement in QoL across both groups. A statistically significant interaction effect between group and assessment was found only for physical functioning (*p* = 0.009, η^2^ = 0.27), indicating that the improvement in this domain over time was more pronounced in the IG compared to the CG. No significant between-group differences were found in any SF-36 domains.

The post hoc analysis showed that, in the IG, Physical functioning domain improved significantly from PreSA to PostSA (*p* = 0.024), and from PreSA to 9MA (*p* = 0.026). While Health changes improved significantly from PreSA to 9MA (*p* = 0.045) and PreSA to 12MA (*p* = 0.012).

In the CG, significant improvements were observed in the Pain domain, with differences between PreSA and 9MA (*p* = 0.005), in Health change domain with differences between PreSA and PostSA (*p* < 0.006), PreSA and 9MA (*p* < 0.001), and PreSA and 12MA (*p* = 0.026).

No statistically significant differences were found in the between-group comparisons at any of the four time points for any of the SF-36 domains.

### 3.2. Clinical Outcomes

The analysis of PROMs showed statistically significant within-subject effects over time for VAS (*p* = 0.005, η^2^ = 0.88) and significant interaction effects between group and time for both VAS (*p* = 0.017, η^2^ = 0.56) and WOMAC (*p* = 0.019, η^2^ = 0.27). A significant interaction effect was also found for the HOOS and KOOS composite score (*p* = 0.031, η^2^ = 0.27), while no significant differences were detected for any sub-score (Supplementary Material—Table S1).

Concerning the VAS, the improvements recorded over time were not statistically significant in the IG. On the contrary, in the CG, significant differences were observed between PreSA and PostSA (*p* = 0.001), PreSA and 3MA (*p* = 0.002), and PreSA and 6MA (*p* < 0.001). No significant between-group differences were found at any follow-up time point.

For WOMAC, no within-group or between-group comparisons were statistically significant.

Regarding HOOS and KOOS, no statistically significant differences were found in the total and their subscales. Descriptive values indicate that both groups followed similar trajectories over time for stiffness, pain, function, sport, and QoL domains.

### 3.3. Strength

The analysis of muscle strength (expressed in kilograms) on both the surgery and non-surgery sides revealed distinct time-related patterns within and between the IG and CG. The strength tests on the surgery leg side revealed statistically significant within-subject effects over time on hip flexion (*p* = 0.040, η^2^ = 0.42), extension (*p* = 0.002, η^2^ = 0.76), and adduction (*p* = 0.038, η^2^ = 0.56) and knee flexion (*p* < 0.001, η^2^ = 0.77) (Supplementary Material—Table S2).

**Figure 3:**
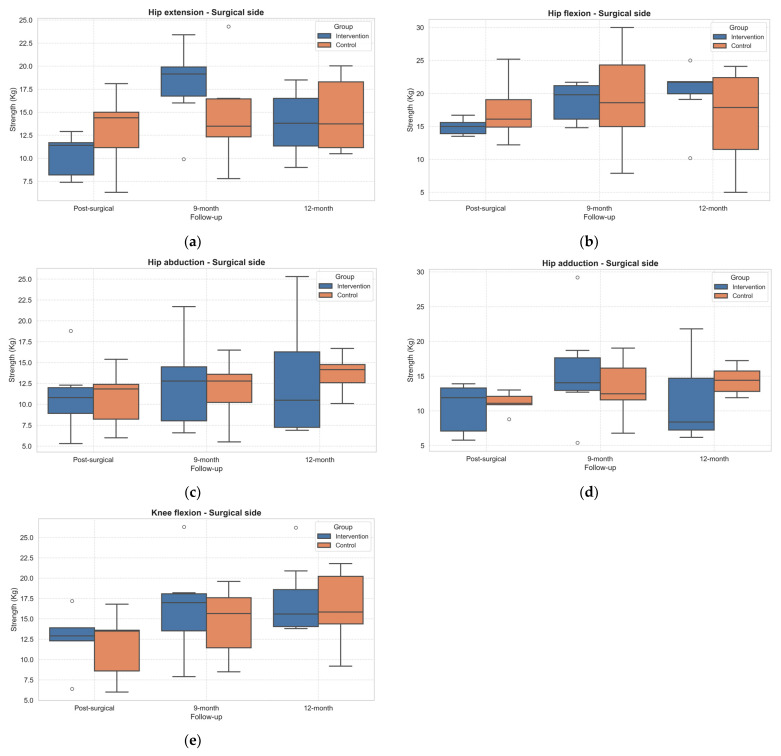
Boxplot of changes in muscle strength across follow-up assessments in the operated limb. (**a**) Hip extension; (**b**) Hip flexion; (**c**) Hip abduction; (**d**) Hip adduction; (**e**) Knee flexion. Outliers, values outside the range of 1.5*IQR, are shown as circles.

In general, all the strength tests improved from PostSA to 9MA in the IG. Not all the improvements were maintained at 12MA (Figure 3). However, no statistically significant improvements emerged from the post hoc analysis in both groups.

### 3.4. Physical Function

**Figure 4:**
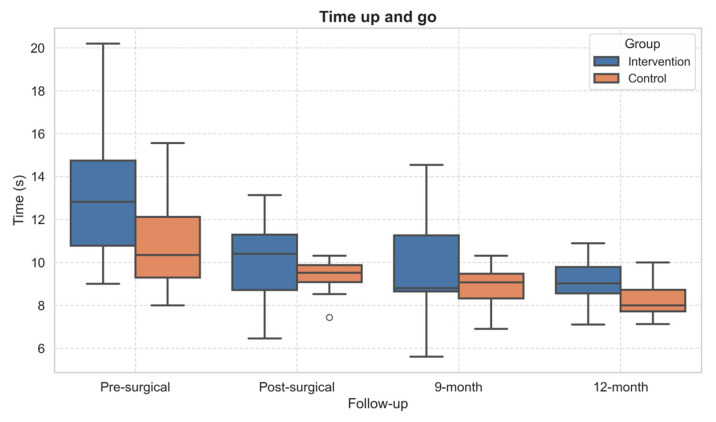
Boxplot of Timed Up and Go test performance across follow-up assessments. Outliers, values outside the range of 1.5*IQR, are shown as circles.

**Figure 5:**
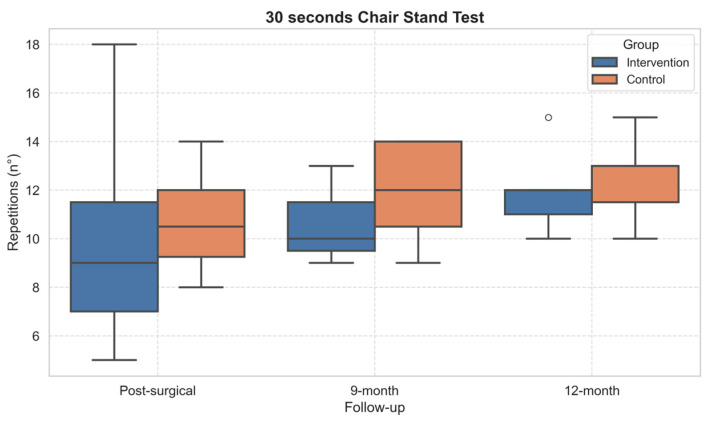
Boxplot of 30-Second Chair Stand Test performance across follow-up assessments. Outliers, values outside the range of 1.5*IQR, are shown as circles.

Concerning the functional tests, statistically significant within-subject effects over time emerged in TUG (*p* = 0.001, η^2^ = 0.57) and 30sCST (*p* = 0.041, η^2^ = 0.29). The performance of both TUG (Figure 4) and 30sCST (Figure 5) improved in both groups over time. However, the post hoc did not show significant differences. 

**Figure 6:**
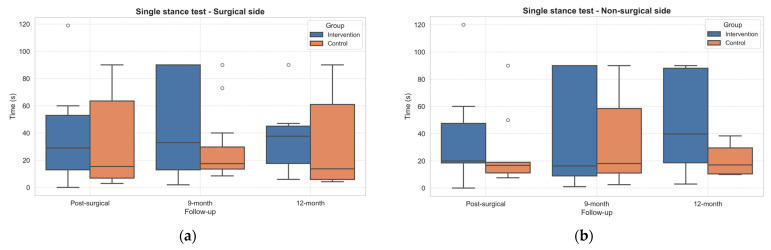
Boxplots of Single Stance Test results (expressed in seconds) on the surgical (**a**) and non-surgical (**b**) sides in both groups across follow-up assessments. Outliers, values outside the range of 1.5*IQR, are shown as circles.

Notably, the single stance test improved sensibly in the IG group with respect to the CG, both in the surgical side and non-surgical side (Figure 6). However, no statistically significant differences emerged.

### 3.5. Adherence to the Intervention

The adherence to a total of 48 exercise sessions for the IG was considerate moderate (63.0 ± 16.1%) [44]. No adverse events were recorded during the intervention.

## 4. Discussion

This pilot RCT evaluated the effectiveness of a specifically adapted PA intervention on the QoL of individuals after THR and TKR. The main result of this study was that, in the IG, the QoL domains of physical functioning and health change improved significantly from PreSA to the 12 months after surgery. This findings aligns with previous studies suggesting that continued engagement in structured PA following standard rehabilitation contributes to enhanced functional recovery and QoL in joint replacement patients [23]. Notably, even the CG showed significant improvements in pain and health changes domains. While this likely reflects the natural trajectory of recovery post-arthroplasty [7], the more pronounced gains in physical functioning in the IG suggest that continuing exercise post-rehabilitation may provide additional value beyond usual care, particularly in domains related to physical capabilities and perceived health improvements. These results also confirm the importance of bridging the gap between rehabilitation discharge and full functional recovery, which is frequently underestimated in clinical practice [20,28]. However, even when statistical significance is observed, it should be acknowledged that the limited sample size may compromise the robustness and generalizability of the findings.

Nevertheless, no significant between-group differences were found in the overall SF-36 scores. Additionally, the non-significant changes observed in the emotional well-being and social functioning domains may suggest that, while PA influences physical health and perception of improvement, its impact on psychological and social domains require either longer follow-up or the integration of complementary psychosocial or behavioral interventions [45,46]. Enhancing these domains require targeted interventions addressing mental health, social support, and lifestyle factors, which were not included in our protocol.

In terms of clinical outcomes, the VAS score improved significantly over time in both groups, with the CG showing notable improvements. This might reflect the efficacy of surgical treatment and early rehabilitation in relieving pain [7]. In contrast, no differences were found in WOMAC, KOOS and HOOS scores. This may partially be explained by the fact that these scales were only administered after surgery, showing that the surgery has notable impact on PROMs improvements. Similar findings have been reported in previous studies assessing clinical outcomes after PA interventions [28]. However, the exercise programs appear to have had a limited impact on the PROMs assessed.

Interestingly, although not statistically significant, individuals in the IG showed clinically relevant improvements in strength after the PA intervention, especially in hip extension, flexion, abduction, and knee flexion in the operated leg. This trend suggests a potential positive impact of the intervention on muscle recovery [8,47]. The absence of significant between-group differences may reflect heterogeneity in patient adherence, baseline functional levels, or unmeasured confounders such as differences in rehabilitation intensity prior to randomization. Additionally, the strength of the CG remained stable or slightly declined over time, underscoring the need for targeted strength training to prevent deconditioning during long-term recovery. Increased muscle strength, especially in the quadriceps and hip abductors, is associated with improved functional outcomes such as walking speed, stair climbing, and daily activities as well as better PROMs and QoL measures, including reduced pain after both TKR and THR [48].

It is important to note that pre-surgical strength assessment was not performed in this study to avoid bias caused by pain-related arthrogenic muscle inhibition, a reflexive decrease in voluntary muscle activation which occurs in the presence of joint pain and swelling, and can significantly distort strength measurements before surgery [49]. However, previous studies reported an approximately 20% to 80% reduction in lower limb strength early after surgery [28,47,50,51], and may take several months or even years to recover. These persistent deficits are not only linked to functional impairment but may also increase the risk of prosthesis loosening and falls if not addressed through adequate interventions [8,47]. Therefore, post-rehabilitation programs should include structured strength training to fully restore pre-surgical functional levels and optimize implant longevity.

It is important to note that evaluating both surgical interventions (THR and TKR) together may have reduced the informative power of these findings. While both TKR and THR significantly improve pain and function, THR patients may report slightly better long-term pain relief and functional outcomes than TKR patients, especially at 5–8 years post surgery [11]. However, other research finds no significant difference in short-term (1 year) PROMs when using comprehensive measures [52].

Regarding functional performance, the TUG, the only test performed also before surgery, showed improvement across follow-up in both groups. This suggests that both standard care and the adapted PA intervention contributed to the recovery of basic daily activities. The observed improvements are consistent with previous studies indicating that TUG scores tend to improve significantly within the first 6–12 months after total joint replacement [26,42].

Moreover, both 30sCST and Single Stance performances improved during follow-ups. Notably, the Single Stance Tests improved more in the IG than the CG, although differences did not reach statistical significance. This finding suggests a potential benefit of the intervention in balance and postural control, which are often impaired after joint replacement due to proprioceptive deficits and changes in joint intracapsular components caused by surgery [53]. Enhancing balance performance is clinically important, as poor balance is a known predictor of fall risk in this population [42].

Overall, adherence to the PA-based intervention program was reported as moderate. This may have affected the clinical and functional outcomes assessed, as low adherence to PA programs consistently results in diminished benefits, including reduced improvements in physical function, metabolic health, and disease management. High adherence is necessary to accurately evaluate the true effect of the intervention [54]. Moreover, psychological factors, in both the cognitive and affective domains, may be important determinants of PA participation and should be considered when designing a PA-based intervention [55].

This study presents some limitations. First, the small sample size limits the statistical power and generalizability of the findings. Hence, the findings should be interpreted with caution and considered preliminary, serving to inform future research with larger cohorts. Second, the distribution of diagnoses was not balanced across the groups, as the CG included mostly participants with THR, while the IG included both THR and TKR. This imbalance may have influenced the results, given potential differences in recovery trajectories and functional outcomes between hip and knee replacements. Third, the rehabilitation phase after surgery was not monitored, and variability in rehabilitation practices may have influenced the observed outcomes. However, assessing patients after surgery and rehabilitation process created a new baseline that was useful to compare the improvements. Finally, the adherence to the intervention was measured only as a percentage of attendance. No additional tools, such as questionnaires or diaries, were used to capture factors influencing participation (e.g., motivation, health issues, or personal barriers). This may have limited our ability to fully understand the reasons behind adherence levels.

## 5. Conclusions

This pilot study supports the feasibility and potential benefits of an adapted PA program following standard rehabilitation in individuals undergoing THR and TKR. Continued exercise contributes to improvements in physical functioning, strength, and balance. Importantly, this study highlights the need for larger, adequately powered trials with long-term follow-up to confirm these findings and refine post-rehabilitation guidelines. Future studies with larger samples, longer follow-up, and additional psychological or behavioral support components could provide a more comprehensive understanding of the multi-dimensional benefits of adapted PA.

## Figures and Tables

**Table 1:** Representative example of an exercise session for individuals with total hip replacement and total knee replacement.

Phase of Exercise Session		Duration	Exercise
Initiation	Warm-up	10 min	- Walk
			- Lateral walking
			- Walk on the forefoot
			- Stepping
			- Scapular adductions
			- Shoulder circumduction
			- Shoulder flection
			- Pelvic anteversion and retroversion
			- Hip circumduction
			- Ankle circumduction
Conditioning	Balance	5 min	- Monopodalic standing
			- Walking on various surfaces
	Strength exercise	35 min	- Squat with fitball/Wall squat
			- Gluteus bridge
			- Hip abduction with elastic band resistance at knee level
			- Adductor isometric contraction
			- Seated knee extension with ankle weight
			- Deadlift
			- Push up against the wall
			- Plank
			- Bent leg raises
Cool down and stretching	Cool down and stretching	10 min	- Hamstring stretching
			- Quadriceps stretching
			- Iliopsoas stretching
			- Triceps surae stretching
			- Trapezius stretching
			- Breathing

**Table 2:** Summary of the instruments used.

Instrument	Administration Method	Assessment Procedure
SF-36	Questionnaire	Self-administered questionnaire during the follow-up
VAS	Visual analogue scale	Self-administered questionnaire during the follow-up
WOMAC	Questionnaire	Self-administered questionnaire during the follow-up
HOOS	Questionnaire	Self-administered questionnaire during the follow-up
KOOS	Questionnaire	Self-administered questionnaire during the follow-up
Handheld dynamometer	Held Hand Dynamometer	Hip flexion: Participants were positioned supine with the knee extended. The dynamometer was placed anteriorly on the thigh above the knee, and participants flexed the hip against resistance Hip extension: Participants were positioned prone with the knee extended. The dynamometer was placed posteriorly on the thigh above the knee, and participants extended the hip against resistance Hip abduction: Participants were seated with hips and knees flexed at 90°. The dynamometer was positioned laterally on the thigh above the knee, and participants abducted the hip against resistance Hip adduction: Participants were seated with hips and knees flexed at 90°. The dynamometer was positioned medially on the thigh above the knee, and participants abducted the hip against resistance Knee flexion: Participants were positioned prone with the knee extended. The dynamometer was placed posteriorly on the leg above the ankle, and participants flexed the knee against resistance
TUG	Physical test	Participants rise from a standard chair, walk 3 m on a flat surface, turn around, walk back, and sit down again as fast as possible (without running)
30sCST	Physical test	Participants were seated on standard chair, with their back straight, feet flat on the floor, and arms crossed over the chest. They rise to a full standing position and return to a seated position as many times as possible within 30 s
Single Stance Test	Physical test	Participants stand on one foot on a flat surface without shoes and maintain this position for as long as possible

**Table 3:** Sample characteristics.

Characteristic		Control Group (*n* = 11)	Intervention Group (*n* = 7)	Statistics Test	*p* Value
		Mean ± SD		*t* test	
Age (y.o.)		66.2 ± 6.4	65.4 ± 8.2	−0.912	0.375
Height (m)		1.7 ± 0.1	1.7 ± 0.1	0.117	0.909
Weight (Kg)		73.6 ± 12.5	73.8 ± 20.5	−1.071	0.301
BMI (Kg/m^2^)		26.0 ± 3.3	25.5 ± 4.1	−2.077	0.055
		Distribution (n°)		Chi^2^	
Surgery type	THR	10	4	2.822	0.093
	TKR	1	3		
Surgery side	Left	3	4	0.595	0.441
	Right	8	3		
Gender	Female	6	5	0.049	0.826
	Male	5	2		
Educational level	Elementary/middle school	2	0	7.148	0.128
	High school	6	5		
	Bachelor/Master degree	3	2		
Occupation	Worker	3	1	2.842	0.242
	Retired	3	3		
Family status	Single	1	1	0.000	1.000
	Married/cohabitant	5	3		

Acronyms: THR, Total Hip Replacement; TKR, Total knee replacement.

**Table 4:** SF-36 domain scores across follow-up time points.

	Control Group (*n* = 11)				Intervention Group (*n* = 7)				Between Subject Effect	Within Subject Effect	
SF-36 Domains	PreSA	PostSA	9MA	12MA	PreSA	PostSA	9MA	12MA	G	A	G*A
Physical functioning	55.0 (45.0)	85.0 (20.0)	92.5 (5.0)	97.5 (20.0)	55.0 (18.8)	**87.5 (12.5) ^a^**	**85.0 (12.5) ^a^**	85.0 (22.5)	0.647	**<0.001**	**0.009**
Role limitations due to physical health	75.0 (81.3)	100.0 (25.0)	100.0 (0.0)	100.0 (0.0)	100.0 (100.0)	100.0 (81.3)	100.0 (0.0)	100.0 (50.0)	0.444	0.071 *	0.876
Role limitations due to emotional problems	100.0 (66.7)	100.0 (8.3)	100.0 (0.0)	100.0 (8.3)	100.0 (25.0)	66.7 (75.0)	100.0 (50.0)	100.0 (0.0)	0.904	0.347	0.082
Energy/fatigue	62.5 (18.8)	62.5 (31.3)	77.5 (23.8)	67.5 (11.3)	57.5 (22.5)	60.0 (5.0)	60.0 (15.0)	65.0 (20.0)	0.956	**0.028**	0.556
Emotional well-being	80.0 (10.0)	84.0 (13.0)	90.0 (12.0)	88.0 (9.0)	70.0 (26.0)	72.0 (16.0)	80.0 (6.0)	72.0 (12.0)	0.661	0.069	0.605
Social functioning	62.5 (37.5)	100.0 (18.8)	100.0 (9.4)	100.0 (0.0)	62.5 (40.6)	87.5 (40.6)	87.5 (25.0)	87.5 (31.3)	0.380	0.105 *	0.953
Pain	45.0 (28.1)	78.8 (35.6)	**100.0 (7.5) ^a^**	100.0 (21.3)	38.8 (48.8)	80.0 (5.0)	77.5 (17.5)	90.0 (37.5)	0.886	**0.001**	0.226
General health	62.5 (36.3)	75.0 (25.0)	77.5 (30.0)	65.0 (16.3)	70.0 (16.3)	65.0 (10.0)	75.0 (15.0)	65.0 (15.0)	0.823	0.116	0.102
Health change	25.0 (0.0)	**75.0 (50.0) ^a^**	**87.5 (25.0) ^a^**	**62.5 (50.0) ^a^**	25.0 (31.3)	75.0 (50.0)	**75.0 (25.0) ^a^**	**75.0 (25.0) ^a^**	0.841	**<0.001**	0.611

Table shows median (IQR). ^a^ Statistically significant improvement compared to PreSA (*p* < 0.05). * Greenhouse-Geisser correction. Bold = *p* < 0.05. Acronymous: PreSA = pre-surgical assessment; PostSA = post-surgical assessment; 9MA = Nine months after surgery assessment; 12MA = Twelve months after surgery assessment.

## Data Availability

The original contributions presented in this study are included in the article/Supplementary Material. Further inquiries can be directed to the corresponding author.

## Supplementary Materials

The following supporting information can be downloaded at: https://www.mdpi.com/article/10.3390/healthcare13182333/s1, Table S1. Time and group comparison of clinical outcome measures. Table S2. Time and group comparison of muscle strength outcomes in hip and knee. Table S3. Time and group comparison of functional performance.

## Abbreviations

The following abbreviations are used in this manuscript:

OA	Osteoarthritis
QoL	Quality of life
THR	Total hip replacement
TKR	Total knee replacement
OECD	Organization for Economic Cooperation and Development
PA	Physical activity
RCT	Randomized controlled trials
PAIR	Physical ActIvity after hip and knee Replacement
IG	Intervention group
CG	Control group
PreSA	Pre-surgical assessment
PostSA	Post-surgical assessment
9MA	Nine months after surgery
12MA	Twelve months after surgery
VAS	Visual Analogue Scale
SF-36	36-Item Short Form Survey
PROM	Patient-reported outcome measures
WOMAC	Western Ontario and McMaster Universities Osteoarthritis Index
HOOS	Hip disability and Osteoarthritis Outcome Score
KOOS	Knee injury and Osteoarthritis Outcome Score
TUG	Timed Up and Go
WHO	World Health Organization
30sCST	30-Second Chair Stand Test
