# Supplementary material for: Effectiveness of Adapted Physical Activity on Quality of Life of Patients with Knee and Hip Replacement: A Randomized Pilot Study
Source: Healthcare (Basel). 2025 Sep 17;13(18):2333. doi: 10.3390/healthcare13182333 (PMC12469286; doi:10.3390/healthcare13182333)
Supplement: Supplementary file 1 [file healthcare-13-02333-s001.zip › healthcare-3789937-supplementary.pdf]

Table S1. ANOVA per repeated measures of clinical outcomes

**PROMs**	Control				Intervention				Between subject effect	Within subject effect	
	PreSA	PostSA	9MA	12MA	PreSA	PostSA	9MA	12MA	G	A	G*A
VAS	5.0 (3.0)	**0.0 (1.3)** [a]	**0.0 (1.0)** [a]	**0.00 (0.50)** [a]	5.50 (2.50)	2.0 (0.5)	2.0 (0.8)	2.0 (1.5)	0.731	**0.005***	**0.017**
WOMAC	-	94.0 (13.7)	98.1 (2.8)	98.4 (3.7)	-	89.1 (19.3)	93.0 (8.2)	84.4 (15.9)	0.114	0.923*	**0.019**
HOOS/KOOS	-	91.6 (14.4)	94.7 (5.1)	95.9 (6.2)	-	74.4 (23.5)	82.0 (9.8)	80.0 (15.9)	0.077	0.898*	**0.031**
• Stiffness	-	82.5 (27.5)	92.5 (11.8)	100.0 (6.5)	-	80.0 (28.0)	86.0 (19.0)	80.0 (25.5)	0.256	0.291*	**0.003**
• Pain	-	98.5 (7.5)	97.5 (2.5)	100.0 (1.2)	-	92.5 (22.8)	92.0 (4.8)	90.0 (18.8)	0.257	0.934*	0.080
• Function	-	96.6 (10.6)	98.8 (1.5)	97.1 (2.6)	-	85.0 (11.8)	96.0 (6.4)	85.3 (15.6)	0.062	0.894*	0.060
• Sport	-	77.5 (12.5)	84.4 (17.9)	85.7 (20.4)	-	56.3 (30.1)	75.0 (15.1)	75.0 (22.5)	0.226	0.894*	0.078
• Quality of life	-	81.3 (9.4)	87.8 (6.3)	87.5 (14.1)	-	56.3 (27.9)	63.0 (9.6)	68.8 (18.8)	0.141	0.804*	0.848

Table S2. ANOVA per repeated measures of strength outcomes

		Control			Intervention			Between subject effect	Within subject effect	
		PostSA	9MA	12MA	PostSA	9MA	12MA	G	A	G*A
Surgery side	Hip extension	14.4 (3.9)	13.5 (4.1)	13.7 (7.2)	11.4 (3.5)	19.2 (3.2)	13.8 (5.2)	0.932	**0.002***	0.120
	Hip flexion	16.1 (4.2)	18.6 (9.3)	17.9 (10.9)	15.0 (1.7)	19.8 (5.1)	21.7 (1.9)	0.716	**0.040**	0.092
	Hip abduction	11.9 (4.2)	12.8 (3.4)	14.2 (2.2)	10.8 (3.1)	12.8 (6.5)	10.5 (9.0)	0.862	0.597	0.498
	Hip adduction	11.1 (1.2)	12.5 (4.6)	14.4 (2.9)	11.9 (6.2)	14.1 (4.7)	8.4 (7.5)	0.767	**0.038**	**0.025**
	Knee flexion	13.5 (5.0)	15.7 (6.2)	15.9 (5.9)	12.9 (1.6)	17.0 (4.6)	15.6 (4.6)	0.773	**<0.001**	0.520
Non surgery side	Hip extension	14.1 (3.0)	15.2 (6.7)	13.1 (9.0)	11.2 (4.4)	19.7 (2.5)	14.5 (6.6)	0.776	**<0.001**	0.464
	Hip flexion	15.3 (6.2)	16.2 (7.4)	16.8 (8.2)	14.4 (3.1)	21.2 (7.9)	20.0 (5.0)	0.787	**0.001**	0.533
	Hip abduction	12.2 (2.9)	13.0 (4.3)	13.8 (4.1)	10.3 (3.7)	13.3 (3.6)	12.3 (6.8)	0.873	0.396	0.688
	Hip adduction	9.5 (2.9)	11.7 (4.3)	13.1 (2.7)	11.6 (0.9)	14.9 (9.7)	11.0 (5.3)	0.448	0.294	0.411
	Knee flexion	9.3 (6.4)	15.0 (4.7)	15.5 (2.3)	12.3 (2.3)	17.0 (11.7)	13.8 (5.9)	0.555	**0.007**	0.888

Note: all the measures are reported as kilograms

Table S3. ANOVA per repeated measures of functional tests

**SF-36**	Control				Intervention				Between subject effect	Within subject effect	
	PreSA	PostSA	9MA	12MA	PreSA	PostSA	9MA	12MA	G	A	G*A
TUG (time, s)	10.3 (2.8)	9.5 (0.8)	9.1 (1.2)	8.0 (1.0)	12.8 (4.0)	10.4 (2.6)	8.8 (2.6)	9.0 (1.2)	0.235	**0.001***	0.057
30sCST (repetitions, n°)	-	10.5 (2.8)	12.0 (3.5)	13.0 (1.5)	-	9.0 (4.5)	10.0 (2.0)	12.0 (1.0)	0.449	**0.041***	0.764
Single stance (time, s)	-	15.4 (56.6)	17.5 (16.1)	13.7 (55.2)	-	29.0 (40.1)	33.0 (77.0)	37.7 (27.4)	0.566	0.977	0.344
Single stance (non-operated leg) (time, s)	-	16.7 (7.8)	18.0 (47.5)	17.0 (19.1)	-	20.0 (29.0)	16.3 (81.0)	39.7 (69.4)	0.291	0.782	0.586
